# A Degree Easily Obtained

**Published:** 1868-05

**Authors:** 


*A Degree Easily Obtained.*—On referring to the catalogue of the Pennsylvania College of Dental Surgery we find that five years of actual practice are considered equivalent to one course of lectures, and that the candidate for graduation in this institution, must show by certificates, that he has been in the actual practice of his profession for the period named, before he can present himself for examination at the end of but one course of lectures. Such being the published rule we wish the profession to learn how strictly it is adhered to, and also how easily the degree of Doctor of Dental Surgery may be obtained at this institution.

A student from Carlisle, Pennsylvania, who has had *less than two years of pupilage* and *no practice*, had conferred upon him at the late commencement of the Penn. College of Dental Surgery the degree of Doctor of Dental Surgery, after attending but one course of lectures.

These facts we are ourselves cognizant of, and can also prove by letters received from gentlemen of the highest character.

One of our correspondents after giving a full history of the *professional life* of the *Doctor of Dental Surgery* referred to, makes use of the following language. "In justice to those who have complied with the requirements of our Colleges, and of those yet contemplating such a course, I deem it a *duty* to acquaint you with the above facts."

We have omitted to notice the proceedings of the late Commencement of the Penn. College in our Journal, for the reason that we are not certain how many others of the graduating class obtained the degree in the same manner; not feeling disposed to acknowledge Doctors of Dental Surgery thus made.

The following extract from a letter received a short time since needs no comment:—"Dear Sir,—I have attended one course of lectures in the Pennsylvania College of Dental Surgery. I went there under the impression that it stood the highest of its kind. I have since learned some facts which if proven true, leave me to place very little value on a diploma coming from that institution. Will you be kind enough to inform me whether, on the strength of that one course, I could be admltted as a second course student at the next term of the Baltimore College."

In an editorial published in the March number of the Journal, it was stated that we had good authority for announcing that the Baltimore College of Dental Surgery had the largest number of students attending the lectures of the past session.

We are not disposed to retract this assertion if regular students only are taken into account. Students are not admitted into the Baltimore College for partial courses, but are required to take out all the tickets, and prove by their attendance on, and attention to the lectures, that they are truly interested in all the branches taught. During the late war the classes of the Baltimore College were necessarily small, for the reason that the greater number of students attending this College are from the Southern States, and owing to the hard struggle through which the institution passed during the time referred to, the return of prosperity is very gratifying to those connected with it.

For this reason the editorial above referred to, in the March number of the Journal, was written.

